# Outcome of Twin Pregnancies Complicated by a Single Intrauterine Death

**DOI:** 10.7759/cureus.26055

**Published:** 2022-06-17

**Authors:** Saleh Al-Alaiyan, Najlaa Abdulaziz, Hanifah Bukhari, Amal Hawari, Amjad Alturki, Reem Alghamdi, Weam Elsaidawi

**Affiliations:** 1 Pediatrics/Neonatal-Perinatal Medicine, King Faisal Specialist Hospital and Research Centre, Riyadh, SAU; 2 Pediatrics, Alfaisal University, Riyadh, SAU; 3 Pediatrics, King Faisal Specialist Hospital and Research Centre, Riyadh, SAU; 4 Obstetrics and Gynaecology, King Faisal Specialist Hospital and Research Centre, Riyadh, SAU; 5 Biostatistics and Epidemiology, King Faisal Specialist Hospital and Research Centre, Riyadh, SAU

**Keywords:** infants, prematurity, co-twin death, twin pregnancy, neonates

## Abstract

Objective

Single intrauterine fetal death (sIUFD) occurs in approximately 6% of twin pregnancies. If it occurs in the second and third trimesters, it places the co-twin at substantial risk, including that of preterm delivery and associated comorbidities of prematurity or neonatal death. The aim of this present study was to determine the outcome of surviving co-twins following spontaneous single intrauterine fetal death.

Methods

This is a retrospective, observational, cohort study that included all twin pregnancies delivered between January 2015 to December 2019 with a gestational age of 24 weeks or more. Maternal data included were: age, medical illnesses, conceivable methods, chorionicity, and complications during pregnancy. Gestational age of intrauterine fetal demise, gestational age of the surviving twin delivery, mode of delivery, and medications used during pregnancy were also recorded*. *Neonatal data included: gestational age, gender, birth weight, Apgar score, and complications of prematurity.

Results

Twenty-two pregnancies were found to be complicated by sIUFD and included in the present study (group 1), compared to 26 twins with no sIUFD (group 2). The incidence of sIUFD in twin pregnancies after 20 weeks of pregnancy was 4.4%. The gestational age (weeks) in group 1 was 34.5 (29-39) and in group 2 was 32 (26-38). The frequency of preterm delivery 81.8% in group 1 (59% monochorionic) and 69.2% (100% dichorionic) in group 2. No significant statistical differences were found between the two groups in complications of prematurity.

Conclusions

We conclude that delaying delivery in twin pregnancies complicated by single intrauterine demise with regular follow-up may lead to delivering infants with fewer complications of prematurity.

## Introduction

Mortality and morbidity have been reported to be more in twin pregnancies in comparison to singleton pregnancies. Single intrauterine fetal death (sIUFD) occurs in approximately 6% of twin pregnancies [1]. First-trimester loss is not known to result in an adverse outcome for the co-twin, although this is controversial. However, sIUFD in the second and third trimesters places the co-twin at substantial risk, including preterm delivery and associated comorbidities of prematurity such as pulmonary hypoplasia, necrotizing enterocolitis, long-term neurological complications, and neonatal death [2-5].

Another possible outcome is the death of the surviving co-twin in utero (following the demise of the first twin) [6]. Literature reports show an increased risk of vascular complications and therefore an increased percentage of visceral and skin damage [7]. On the other hand, damage to the central nervous system is characteristic of the third trimester. In addition, there are increased risks to the mother of pre-eclampsia, coagulopathy, and sepsis [8].

Recent researchers proved that the demise of one twin occurs after 24 weeks of gestation in 1.1% of dichorionic twins compared to 3.6% of monochorionic twins [9]. On the other hand, this risk is higher before 24 weeks of gestation and its prevalence is 12.2% for monochorionic pregnancy and 1.8% for dichorionic pregnancy [1].

Monochorionic pregnancies are at greater risk due to their shared placental circulation [2, 3]. Twin-twin transfusion syndrome (TTTS) is the commonest major complication of monochorionic twin pregnancy and results in significant perinatal morbidity and mortality [10-12]. The aim of this study was to determine the outcome of surviving co-twins following spontaneous single intrauterine fetal death.

## Materials and methods

This is a retrospective, observational, cohort study that was conducted in the Neonatal Intensive Care Unit (NICU) at King Faisal Specialist Hospital in Riyadh. The research proposal has been reviewed and approved by the Research Advisory Council of our hospital (RAC number: 2191248). All required data were retrieved from the hospital integrated clinical information system (ICIS) and medical charts of women and infants. From January 2015 to December 2019, all twin pregnancies with a gestational age of 24 weeks or more were included. Twin pregnancies were excluded from the study if the antenatal ultrasound showed twin reversed arterial perfusion sequence and or chromosomal abnormalities confirmed by chorionic villus sampling or amniocentesis.

Maternal data included in our study were: age, medical illnesses, methods of conception (spontaneous pregnancy or assisted reproductive technology), chorionicity, and complications during pregnancy which included infection, TTTS, abnormal placenta and disseminated intravascular coagulation (DIC). Information about the gestational age of intrauterine fetal demise, gestational age of the surviving twin delivery, mode of delivery, and medications used during pregnancy such as cortisone and magnesium sulphate were collected. Twin-twin transfusion syndrome (TTTS) was diagnosed with ultrasound and the syndrome was defined as a monochorionic diamniotic pregnancy in which there was polyhydramnios (maximum vertical pocket of > 8 cm) in one amniotic sac and oligohydramnios (maximum vertical pocket of < 2 cm) in the other. In addition to the disparate volumes of amniotic fluid, the staging was based on several other variables, including the appearance of the donor fetus’s bladder, abnormal umbilical blood flow as measured by Doppler ultrasound, presence or absence of hydrops, and the death of one twin. Using these variables, TTTS was staged by Quintero et al [13] from a score of I (mild) to V (severe).

Neonatal data included: gestational age, gender, birth weight, Apgar score, the use of inotropes for hypotension, blood transfusion, umbilical cord gas, intrauterine growth restriction (IUGR), renal injury, brain imaging, anomalies secondary to vascular disturbance, complications of prematurity (respiratory distress syndrome [RDS], necrotizing enterocolitis [NEC], intraventricular hemorrhage [IVH], periventricular leukomalacia [PVL], and sepsis).

The medical conditions used in this study were defined as follows: Single intrauterine fetal demise (sIUFD): death at or after 20 weeks of gestation; Monochorionic twins: identical twins who share one placenta; Dichorionic twins: each having their own placenta and amniotic fluid; Monochorionic monoamniotic twins: identical twins who share both a placenta and an amniotic sac; Monochorionic diamniotic twins: identical twins who share a placenta but not an amniotic sac; A premature birth was defined as neonates born alive before 37 weeks of pregnancy are completed; Intrauterine growth restriction (IUGR) was defined as growth at the 10th or less percentile for the weight of the fetus at that gestational age; Metabolic acidosis was defined as a base deficit of -4 mEq/L and more, pH equal to or below 7.20, and bicarbonates below 15 mEq/L; and Hypotension was defined as any value that falls below the 5th or 10th percentile for gestational and postnatal age.

Statistical method

For continuous variables, the Wilcoxon Rank Sum test was used. Fisher Exact test was used for categorical variables. Continuously scaled data were presented as medians and interquartile ranges and count data were presented as numbers and percentages. A p-value of less than 0.05 was considered statistically significant.

## Results

During the five-year study period, 227 twin pregnancies were identified. Of these 22 pregnancies were complicated by single intrauterine demise and were included in the present study (group 1). These infants were compared to members of twins (26 newborns) born to 13 mothers during the same period and matched by the birth weight, and gestational age (group 2). Twenty of group 1 (90.9%) had fetal demise after 20 weeks of pregnancy and two had it at 20 (9.1%) weeks of pregnancy. Accordingly, in this present study, the incidence of a single fetal death in twin pregnancies after 20 weeks of pregnancy was 4.4%.

The median age of mothers (in years) was 28 (24.75-34.25) for group 1 and 32 (30.5-37) for group 2, with a p-value of 0.0576. In group 1, five (22.7%) of the mothers were primiparous, and 17 (77.3%) were multiparous, while in group 2, 10 (38.5%) and 16 (61.5%) were primiparous and multiparous, respectively (Table 1).

**Table 1:** Maternal and fetal characteristics in the two groups *Median (range), ART=assisted reproductive technology, TTTS=twin-twin transfusion syndrome, IUFD=intrauterine fetal death

	Group 1 (N=22)	Group 2 (N= 26 infants of 13 mother)	P Value
Maternal age (years)	28 (24.75, 34.25) *	32 (30.5, 37)	0.0576
Primiparous	5 (22.7%)	10 (38.5%)	
Multiparous	17 (77.3%)	16 (61.5%)	
Spontaneous pregnancy:	17 (77.27%)	8 (61.54%)	
Pregnancy achieved by ART	5 (22.7%)	5 (19.2%)	
Antenatal cortisone	14 mothers (63.64%)	5 (38.46%)	0.1789
TTTS	6 (27.27%)	(7.69%)	
TTTS managed by Amnioreduction laser ablation, blood transfusion	19 (86.36%)	1 (7.69%)	
Dichorionic	9 (40.90%)	20 (76.92%)	
Monochorionic	13 (59.09%)	6 (23.07%)	
Caesarean section	16 (72.72%)	26 (100%)	
Gestation at diagnosis of IUFD (weeks)	28 (20-36)	0%	
IUFD diagnosed beyond 20 weeks	20 (90.9%	0%	
Abnormal antenatal ultrasound in amniotic fluid, growth velocity, and Doppler findings	12 (54.55%)	6 (46.15%)	0.7332

Pregnancy was achieved spontaneously in 17 of 22 (77.27%) in group 1 and eight of 13 (61.54%) in group 2, with a statistically non-significant p-value (0.4437), and with assisted reproductive technology (ART) was five in both groups. There were 14 mothers (63.64%) in group 1 and five in group 2 (38.46%) who received cortisone antenatally, with a statistically non-significant p-value (0.1789). Twin-twin transfusion syndrome (TTTS) was confirmed in six of 22 (27.27%) in group 1 and one of 13 (7.69%) in group 2. Amnioreduction, laser ablation alone and laser and blood transfusion were performed in 15 of 22 (68.18%) in group 1 and one of 13 (7.69%) in group 2. Abnormal antenatal ultrasound findings in the form of oligohydramnios, polyhydramnios, IUGR, doppler findings were found in 12 of 22 (54.55%) of group 1 and six of 13 (46.15%) of group 2 with a p-value of 0.7332. There were nine dichorionic/diamniotic (Di/ Di), 11 monochorionic/diamniotic (Mono/Di), and two monoamniotic/monochorionic (Mo/Mo) in group 1, while 20 Di/ Di, two Mono/Di and four Mo/Mo in group 2. Caesarean section was performed in 16 (72.7%) of group 1 and 26 (100%) in group 2. Maternal diseases - gestational diabetes (group 1=1 and group 2 =4), antiphospholipid syndrome (group 1=1 and group 2= 2), intrauterine growth restriction (group 1=9 and group 2=8), preeclampsia - were found in three mothers in group 1 and there was no evidence in the two groups in terms of bleeding, DIC, and infection. There were six (27.3%) males and 16 (72.7%) females in group 1 and 11 (42.3%) males and 15 (57.7%) females in group 2. The median (range) of gestational age (weeks) in group 1 was 34.5 (29-39) and in group 2 it was 32 (26-38), with a p-value of 0.62414.

The frequency of preterm delivery was 81.8% in group 1 (59% monochorionic) and 69.2% (100% dichorionic) in group 2; the difference was statistically not significant (p-value 0.5048) (Table 1).

The median (range) of birth weight (grams) in group 1 was 1860 (1402.5-2367.5) and in group 2 was 1800 (1552.5-2450), with a p-value of 0.992. The median (range) of Apgar score at 5 minutes for groups 1 and 2 was 9 (8-10) and 8 (7- 9), respectively with a p-value of 0.083. There were no significant statistical differences between the two groups in the neonatal complications such as RDS, NEC, sepsis, hypotension, brain injury (one infant, 29 weeks’ gestation, developed grade 3 IVH, and one infant, 26 weeks’ gestation, developed PVL), blood transfusion, metabolic acidosis, and acute kidney injury (Table 2). All the surviving twins and their controls were discharged home in good conditions.

**Table 2:** Demographics, complications and outcome of infants in the two groups *Median (range), RDS=respiratory distress syndrome, NEC=necrotizing enterocolitis, IVH=intraventricular hemorrhage, PVL= periventricular leukomalacia

	Group 1 (N=22)	Group 2 (N= 26 infants of 13 mother)	P Value
Gestational age (weeks)	34.5 (29-39)*	32 (26-38)	0.62414
Gender (Males)	7 (31.8%)	10 (38.46%)	0.1025
Birth weight (grams)	1860 (1402.5-2367.5)	1800 (1552.5-2450)	0.992
Apgar score at 5 minutes	9 (8-10)	8 (7-9)	0.083
RDS	5 of 22 (22.7%)	4 of 26 (15.4%)	0.7131
NEC	2 of 22 (9%)	2 of 26 (7.7%)	1.00
IVH	1 (grade 3)	0	
PVL	1	0	
Hypotension	2 (9.1%)	1 (3.9%)	0.5866
Blood transfusion	4 o (18.2%)	3 (11.54%)	0.6873
Metabolic acidosis	2 (9.1%)	1 (3.9%)	0.5866
High creatinine	1 (4.6%)	1 (3.9%)	1.00

## Discussion

Twins have higher mortality and morbidity than singletons and the risk is higher among surviving co-twin of a demised fetus [1]. At particular risk among twins are monozygotic twins who have poorer survival rates than dizygotic twins [14]. A published study reported that the frequency of mortality in the surviving fetus was 15% in monochorionic twin pregnancies and 3% in dichorionic twin pregnancies [15]. The morbidity and mortality rates seen are attributed to premature delivery, vascular anastomoses between twins, congenital anomalies, and knotting of the umbilical cords [16].

Maternal disseminated intravascular coagulopathy has been previously reported [17] following single intrauterine fetal demise; in our study, we had no case of DIC in either of the two groups.

Our study aimed, primarily, to review the risk of morbidity in the surviving twin when the co-twin died in utero. We found that 92% of surviving twins were monochorionic in comparison to their control who were all dichorionic. Moreover, neonatal mortality was not observed in either group, this is likely to be related to the small sample size. Twin-twin transfusion syndrome is considered the leading cause of single fetal death [18]. In this present study, TTTS was found in 27.27% of the patient group while in the control was 7.69%. The frequency of preterm delivery was 81.8% in group 1 (59% monochorionic) and 69.2% (100% dichorionic) in group 2.

When the death of a monochorionic twin is diagnosed in the third trimester, some obstetricians would like to have the co-twin delivered to avoid complications such as disseminated intravascular coagulation. On the other hand, early delivery might add risks of prematurity to survivors. There is now clear evidence that the early pregnancy loss in twin gestation, the vanishing twin phenomenon, does not have any adverse effect on the course of pregnancy [19, 20]. Thus, the decision of delivering the fetus will depend on its viability and its reasonable chance of survival. On the other hand, single fetal demise after 20 weeks of gestation is rare. In the present study, the incidence of a single fetal death in twin pregnancies after 20 weeks of pregnancy was 4.4%. This is in agreement with the published data which showed that the overall incidence among all twin pregnancies ranges from 2.6% to 6.2% [21]. In the present study, we found no significant statistical differences in the rates of complications in the surviving twin following the intrauterine fetal demise in terms of brain injury (IVH and PVL), RDS, NEC, metabolic acidosis, hypotension, and acute kidney injury. In a meta-analysis [12] that included 39 studies, authors reported that the rate of abnormal antenatal brain imaging was one in five of surviving monochorionic co-twins. This may increase the risk of cerebral impairment in these live-birth co-twins, and this risk has been estimated to be 20% (95% CI 16-25). Moreover, twin-to-twin transfusion syndrome (TTTS) and intrauterine death of a co-twin are associated with neurodevelopmental delay specifically in monochorionic twins. In a case-control study of a regional cohort of 52 twins with TTTS born in 1992-99 in Western Australia, authors found that TTTS had statistically and clinically significant reductions in intelligence scores at 3-6 years of age when compared with their respective control groups [22]. The prevalence of major neurologic deficiencies with TTTS who underwent laser ablation has been reported to be 11% [23].

In our study, only one case of grade 3 IVH in the group of surviving co-twin and one case of PVL in the control group were identified. This was likely because pregnancies continued to a gestational age with a better chance to have much less severity in the complications of prematurity. The medians of gestational age, when infants were born, were 34 and 32 weeks gestation in groups 1 and 2, respectively. There is adequate evidence that infants with lower gestational age are associated with an increased risk of neurodevelopmental disability. The present study did not include the neurodevelopmental assessment of all infants to exclude the effect of prematurity on their cognitive outcome. We did not find any statistically significant differences between the two groups in the complications of prematurity.

The main limitation of this present study is the relatively small number of recruited patients to adequately explore the fetal and maternal complications that are associated with single intrauterine death.

## Conclusions

We conclude that delaying delivery in twin pregnancies complicated by a single intrauterine demise with regular follow-up may lead to delivering infants with fewer complications of prematurity. More prospective trials are required to determine the obstetric and neonatal strategies for managing single fetal death in twin pregnancies. Additionally, to have more data to compare the outcome of infants born to monochorionic and dichorionic pregnancies.

## References

[1] Pharoah P, Adi Y (2000). Consequences of in-utero death in a twin pregnancy. Lancet.

[2] Hillman SC, Morris RK, Kilby MD (2010). Single twin demise: consequence for survivors. Semin Fetal Neonatal Med.

[3] Shek NW, Hillman SC, Kilby MD (2014). Single-twin demise: pregnancy outcome. Best Pract Res Clin Obstet Gynaecol.

[4] Mackie FL, Morris RK, Kilby MD (2016). Fetal brain injury in survivors of twin pregnancies complicated by demise of one twin: a review. Twin Res Hum Genet.

[5] Quarello E, Molho M, Ville Y (2007). Incidence, mechanisms, and patterns of fetal cerebral lesions in twin-to-twin transfusion syndrome. J Matern Fetal Neonatal Med.

[6] Giwnewer U, Wiznitzer A, Friedler JM, Sergienko R, Sheiner E (2012). Intrauterine fetal death of one twin of diamnionic twins is associated with adverse perinatal outcome of the co-twin. J Matern Fetal Neonatal Med.

[7] Bajoria R, Wee LY, Anwar S, Ward S (1999). Outcome of twin pregnancies complicated by single intrauterine death in relation to vascular anatomy of the monochorionic placenta. Hum Reprod.

[8] Glinianaia SV, Pharoah PO, Wright C, Rankin JM (2002). Fetal or infant death in twin pregnancy: neurodevelopmental consequence for the survivor. Arch Dis Child Fetal Neonatal Ed.

[9] Haque R, Rahman MM, Akhter K (2018). Intrauterine single fetal demise in twin pregnancy. Delta Medical College Journal.

[10] Cincotta RB, Gray PH, Phythian G, Rogers YM, Chan FY (2000). Long term outcome of twin-twin transfusion syndrome. Arch Dis Child Fetal Neonatal Ed.

[11] Wagner S, Repke JT, Ural SH (2013). Overview and long-term outcomes of patients born with twin-to-twin transfusion syndrome. Rev Obstet Gynecol.

[12] Mackie FL, Rigby A, Morris RK, Kilby MD (2019). Prognosis of the co-twin following spontaneous single intrauterine fetal death in twin pregnancies: a systematic review and meta-analysis. BJOG.

[13] Quintero RA, Morales WJ, Allen MH, Bornick PW, Johnson PK, Kruger M (1999). Staging of twin-twin transfusion syndrome. J Perinatol.

[14] Myrianthopoulos NC (1970). An epidemiologic survey of twins in a large, prospectively studied population. Am J Hum Genet.

[15] Hillman SC, Morris RK, Kilby MD (2011). Co-twin prognosis after single fetal death: a systematic review and meta-analysis. Obstet Gynecol.

[16] Petersen IR, Nyholm HC (1999). Multiple pregnancies with single intrauterine demise. Description of twenty-eight pregnancies. Acta Obstet Gynecol Scand.

[17] Mitra AG, Chescheir NC, Cefalo RC, Tatum BS (1993). Spontaneous resolution of hypofibrinogenemia in a triplet gestation associated with second trimester in utero death of two fetuses. Am J Perinatol.

[18] Aslan H, Gul A, Cebeci A, Polat I, Ceylan Y (2004). The outcome of twin pregnancies complicated by single fetal death after 20 weeks of gestation. Twin Res.

[19] Zamani Z, Parekh U (2022 Jan-). Vanishing twin syndrome. StatPearls [Internet].

[20] Yaman Tunç S, Ağaçayak E, Yaman Görük N (2015). Single intrauterine demise in twin pregnancies: Analysis of 29 cases. Turk J Obstet Gynecol.

[21] Ong SS, Zamora J, Khan KS, Kilby MD (2006). Prognosis for the co-twin following single-twin death: a systematic review. BJOG.

[22] Dickinson JE, Duncombe GJ, Evans SF, French NP, Hagan R (2005). The long term neurologic outcome of children from pregnancies complicated by twin-to-twin transfusion syndrome. BJOG.

[23] Banek CS, Hecher K, Hackeloer BJ, Bartmann P (2003). Long-term neurodevelopmental outcome after intrauterine laser treatment for severe twin-twin transfusion syndrome. Am J Obstet Gynecol.

